# Liquor Sennæ

**Published:** 1875-09

**Authors:** 


Liquor Sennæ.—Mr. Percy Wells (*Phar. Jour.*) speaks very highly of the convenience and efficiency of the following preparation. The formula has been in use for a period of twenty-seven years, and the preparation afforded has never failed as an active but non-griping purgative: Thirteen ounces of water, three ounces of rectified spirit, and thirty minims of liquor potassæ are mixed and poured upon six ounces of small, sifted, Alexandrian senna. The vessel, or jar, containing the mixture is covered, and the contents stirred once or twice daily for seven days, submitted so strong pressure, and the liquor strained through fine muslin or calico. In about a week a slight deposit forms, which may be separated, when the liquor will remain permanently clear. One part of this, mixed with three parts of water, forms a preparation similar to the officinal infusion of senna.—*Phar. Gaz.*

